# The Use of Biocompatible Membranes in Oral Surgery: The Past, Present & Future Directions. A Narrative Review

**DOI:** 10.3390/membranes12090841

**Published:** 2022-08-29

**Authors:** Ioannis Kormas, Alessandro Pedercini, Hatem Alassy, Larry F. Wolff

**Affiliations:** 1 Department of Periodontics, School of Dentistry, Texas A&M University, Dallas, TX 75246, USA; 2 Centro Odontostomatologico Daina, 24027 Nembro, Italy; 3 Private Practice, San Diego, CA 92064, USA; 4 Division of Periodontology, Department of Developmental and Surgical Sciences, School of Dentistry, University of Minnesota, Minneapolis, MN 55455, USA

**Keywords:** membranes, alveolar ridge preservation, sinus floor augmentation, guided tissue regeneration, guided bone regeneration

## Abstract

The use of biocompatible membranes in periodontal and oral surgery is an important part of regeneration. Over the years, several different membranes have been developed, ranging from non-resorbable membranes that have to be removed in a separate procedure, to collagen membranes that completely resorb on their own, thus avoiding the need for a second surgery. Autogenous membranes are becoming increasingly popular in more recent years. These membranes can be used with a great variety of techniques in the four main hard tissue regenerative procedures: guided tissue regeneration, alveolar ridge preservation, guided bone regeneration and sinus floor augmentation. A review of the literature was conducted in order to identify the most commonly used membranes in clinical practice, as well as the most promising ones for regeneration procedures in the future. The information provided in this review may serve as a guide to clinicians, in order to select the most applicable membrane for the clinical case treated as the correct choice of materials may be critical in the procedure’s success.

## 1. Introduction

The regeneration of the periodontium and deficient ridges has always been one of the most challenging goals in the field of periodontology. The principle of these procedures depends on the separation of the graft from the adjacent tissues, in order for the bone graft to regenerate [1]. Regeneration occurs as a result of a combination of techniques and materials. One of the “key” materials in regeneration are biologic membranes.

There are several membranes currently available. The membranes that were more popular in the early years of regeneration procedures were fabricated from expanded polytetrafluoroethylene (ePTFE). Currently, there is a great variety of non-resorbable membranes, including dense PTFE (dPTFE) and Titanium Mesh (Ti-Mesh) membranes. Membranes like these are sturdier and retain the shape given to them by the operator at the time the regeneration procedure is performed. However, they must be removed in a second surgical procedure.

The collagen resorbable membranes have increased in popularity in recent years, considering that the clinical results are comparable with those achieved with the non-resorbable membranes. While collagen membranes have the advantage of not requiring a second surgical procedure, they do not retain their shape to the extent non-resorbable membranes do, making their use in more extensive surgeries more complicated. Recently, platelet-rich fibrin (PRF) autogenous membranes have been utilized in regenerative procedures [2]. However, little support exists in the literature for the use of such membranes with the same predictability as the previously mentioned membranes.

The purpose of this manuscript is to critically appraise the literature and present a variety of membranes used in periodontal and oral surgery procedures, with the hope that this will serve as a guide for clinicians to select the correct membrane using evidence-based data.

## 2. Guided Tissue Regeneration

Periodontitis is a chronic inflammatory disease affecting the tooth-supporting tissues, including the alveolar bone, cementum and periodontal ligament [3]. Periodontal surgeries attempt to regenerate these lost structures in specific situations such as intrabony and furcation defects. Regeneration is the rebuilding of the lost structures and replacement of their original shape and function [4].

Following periodontal flap surgery, long junctional epithelium forms on the instrumented root surface thus preventing a new connective tissue attachment. This is due to the rapid apical migration of epithelial cells. Regenerative surgeries aim to guide epithelial attachment to a coronal position which allows bone/cementum and new periodontal ligament to re-form on the root surface. The guide used is a physical barrier, typically a membrane, which excludes the microflora and gingival epithelial cells from disturbing the blood clot which adheres on the root surface [4,5]. This concept of guided tissue regeneration (GTR) was first introduced by Nyman in 1982 using a millipore filter as a barrier membrane [6]. The ideal membrane used in GTR should have the following properties: (A) biocompatible without causing inflammatory reactions, (B) undergo degradation matching new regenerated tissue formation, (C) physically adequate to be properly placed and avoid collapse and act as a barrier [7]. 

Regeneration of furcation defects has been studied in the literature and several different techniques and materials have been used; however, multiple other factors must also be assessed when regeneration is considered as a treatment option. In 1995, Machtei and Schallhorn proposed a decision tree regarding furcation regeneration that comprehensively examines factors that could adversely affect the treatment outcome [8]. Camelo in 2000 achieved 89% success in furcation regeneration when using autogenous bone graft under an ePTFE membrane [9]. Non-resorbable membranes have good mechanical properties, are inert, biocompatible, and allow space for new tissue formation. However, as previously mentioned, these membranes are not degradable and require an additional surgical procedure for removal. 

**Figure 1:**
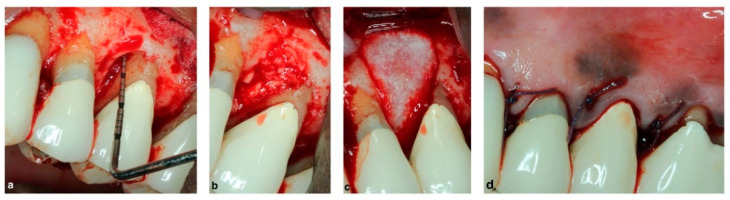
Guided tissue regeneration using a resorbable membrane: (**a**) soft tissue flap reflected, intra-osseous defect; (**b**) bone graft placed in intra-osseous defect; (**c**) resorbable collagen membrane placed over bone graft; and (**d**) soft tissue flap placed and sutured over membrane.

Resorbable membranes, on the other hand, do not need an additional surgery for membrane removal. Guided Tissue Regeneration aided by the use of a resorbable collagen membrane is shown in Figure 1. Synthetic (polylactic acid or copolymers of polylactic acid and polyglycolic acid) membranes are biocompatible, bio-degradable and easy to clinically handle. Initially, they demonstrate high strength, but they lose their structural properties within weeks which may limit their use. Collagen-based membranes are biocompatible; however, their degradation and mechanical properties are unpredictable and they are expensive. A cross-linking agent is often used to enhance their mechanical stability, yet a recent systematic review concluded that cross-linked membranes present higher rates of post-operative complications [10]. Collagen membranes commonly used in dentistry are derived from porcine or bovine sources [1]. These membranes are fibrous proteins, typically collagen Type I and III. The resorption time ranges between 4 and 36 weeks, according to the manufacturers. It is worth noting that a brand of type I collagen membrane is derived from human cadaver skin (Alloderm, BioHorizons, Birmingham, AL, USA) and is typically used in dentistry for root coverage procedures. Resorbable polyglactin membranes have been compared with non-resorbable membranes in class 1 and 2 furcation defects [11]. The authors concluded that similar positive 5-year regeneration clinical results were achieved with either membrane type.

Medical grade calcium sulfate (CS) has been compared with ePTFE membranes in GTR of intrabony defects [12]. CS was both mixed with demineralized freeze-dried bone allograft (DFDBA) and subsequently used as a barrier while osseous defects in the control group were treated with DFDBA and covered with ePTFE. It was concluded that test and control sites did not differ significantly in defect fill and resolution. Therefore, CS showed promise as a barrier instead of a membrane. 

PRF for the treatment of periodontal intrabony defects has been recently studied in a systematic review [13]. The use of PRF in combination with open flap debridement (OFD) significantly improved pocket depth (PD), clinical attachment level (CAL), and bone fill, showing comparable outcomes to the combination of OFD and bone graft. Another meta-analysis reported that PRF had favorable results when used for surgical regeneration procedures in periodontal defects. PRF can be condensed into the defect for space maintenance as well as to cover the defect similarly to a GTR membrane [14]. 

Amniotic membranes have also recently been used in dentistry. A recent study evaluated the effect of using amniotic membranes over DFDBA in infrabony defects. These authors concluded that this membrane did not achieve a statistically significant difference when compared to DFDBA alone [15]. 

Enamel Matrix derivative (EMD) has been shown to enhance periodontal regeneration when used alone after OFD [16]. However, in another investigation the results improved with the addition of a bone graft with EMD [17]. Early wound healing is essential after such regenerative procedures, yet there is still no consensus whether GTR or EMD show better clinical regeneration results [18]. 

Regenerative surgery of class II furcations demonstrated clinical improvement (furcation closure or the conversion to a class I defect which has a more favorable prognosis) for the majority of defects compared with OFD [19]. In this systematic review and meta-analysis, treatment modalities involving a bone graft were associated with a more favorable clinical performance even without a membrane. The use of non-resorbable and resorbable membranes led to similar improvements, while the use of EMD resulted in less post-operative pain and swelling. The review did not find a gold standard as a treatment modality for class II furcations. This review, however, stressed that the studied furcation defects were mainly in mandibular molars rather than maxillary defects, where proximal defects of maxillary molars may be more challenging to treat. 

Incorporating biomaterials into resorbable membranes is showing promise. An angiogenic small molecule, dimethyloxalylglycine, and an osteoinductive inorganic nanomaterial, nanosilicate, were incorporated into a fibrous poly (lactic-co-glycolic acid) membrane which was successfully used for periodontal regeneration on rats [20]. Other biomaterials have also recently been incorporated within resorbable membranes with varying clinical results. Zinc-loaded membranes permit cell viability and promote mineral precipitation in vitro [21]. Recently, an in vitro study showed that doxycycline incorporated into polymeric membranes improved the proliferation and differentiation of osteoblasts [22].

## 3. Guided Bone Regeneration

### 3.1. Alveolar Ridge Preservation

Alveolar ridge preservation (ARP) is a surgical procedure aimed to maintain the ridge height and width of a site after an extraction [23]. Several publications are in support of this procedure for preserving alveolar ridge bone compared with an extraction alone. Socket grafting has been shown to preserve the alveolar ridge width, and to a lesser extent, height [24]. It has also been associated with less frequent need for additional grafting at the time of implant placement [25]. However, it has not been objectively linked to improved implant survival and success rates or less prominent marginal peri-implant bone level changes.

A wide variety of dental materials have been utilized for alveolar ridge regeneration procedures, with biologic membranes being a category of materials commonly used. However, most articles in the literature focus on the selection of bone grafts or technique [23,24,26]. The superiority of one technique to achieve optimal clinical results has still not been established [24]. 

Only a few articles assess the presence and selection of a membrane in ARP procedures. In a recent systematic review and meta-analysis by Bassir et al. the use of a barrier membrane led to improved outcomes in an ARP procedure compared with when no membrane was used [27]. This study did not differentiate among various types of membranes used in the included studies. On the other hand, these authors evaluated the data based on the presence or absence of barrier membranes. Other manuscripts support that the presence of a barrier membrane alone is sufficient [28].

**Figure 2:**
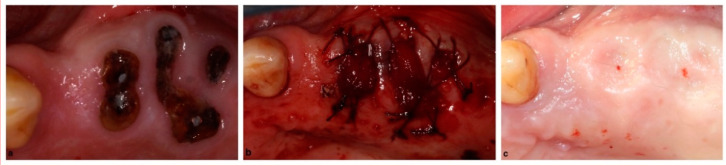
Alveolar ridge preservation with allogenic bone graft and collagen sponge: (**a**) extraction sites of posterior maxillary teeth, (**b**) bone graft placed into socket covered with collagen sponge, (**c**) extraction site healed with excellent keratinized gingival tissue with ideal dimensions preservation.

**Figure 3:**
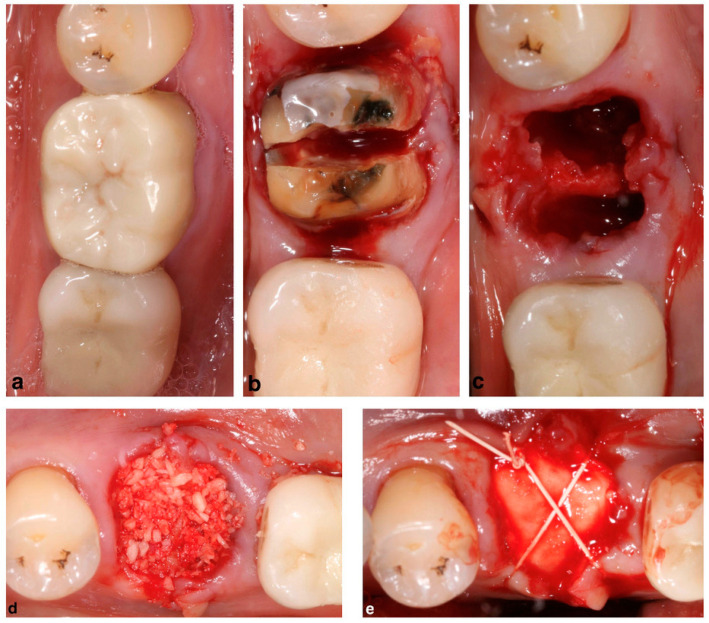
Alveolar ridge preservation with allogenic bone graft and collagen membrane: (**a**) mandibular molar to be extracted; (**b**) mandibular molar sectioned to allow an atraumatic extraction, preserving the alveolar bone, especially on the buccal and lingual aspect; (**c**) molar successfully extracted, preserving the buccal and lingual bone, as well as the septum between the roots; (**d**) freeze-dried bone allograft placed in the socket; and (**e**) collagen resorbable membrane placed over the bone graft and sutured to secure the placement of the membrane and approximate the buccal and lingual soft tissue.

**Figure 4:**
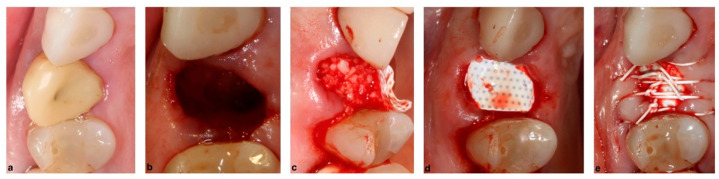
Alveolar ridge preservation with allogenic bone graft and a non-resorbable dPTFE membrane: (**a**) maxillary right canine prior to extraction, (**b**) canine extraction site, (**c**) freeze-dried bone allograft placed in the socket, (**d**) dPTFE non-resorbable membrane placed over bone graft, and (**e**) sutures placed to secure membrane and bone graft.

The current preference for alveolar ridge preservation is an allogenic or xenogenic type of bone graft with a resorbable collagen sponge (Figure 2) or a resorbable collagen membrane (Figure 3). Regarding the use of a collagen membrane, some articles have even researched the effect of a double collagen layer, with no clinically significant difference from using a single layer of collagen membrane [29]. While the most commonly used membrane is a resorbable membrane, non-resorbable membranes have been utilized and reported in the literature with promising results [30,31,32,33] (Figure 4). In a comparison between ARP procedures with collagen sponge or a non-resorbable membrane, the dimensions of the bone are maintained, and the amount of vital bone is similar between the two regeneration procedures, which would make the collagen sponge a cheaper and less technique sensitive alternative [34]. 

**Figure 5:**
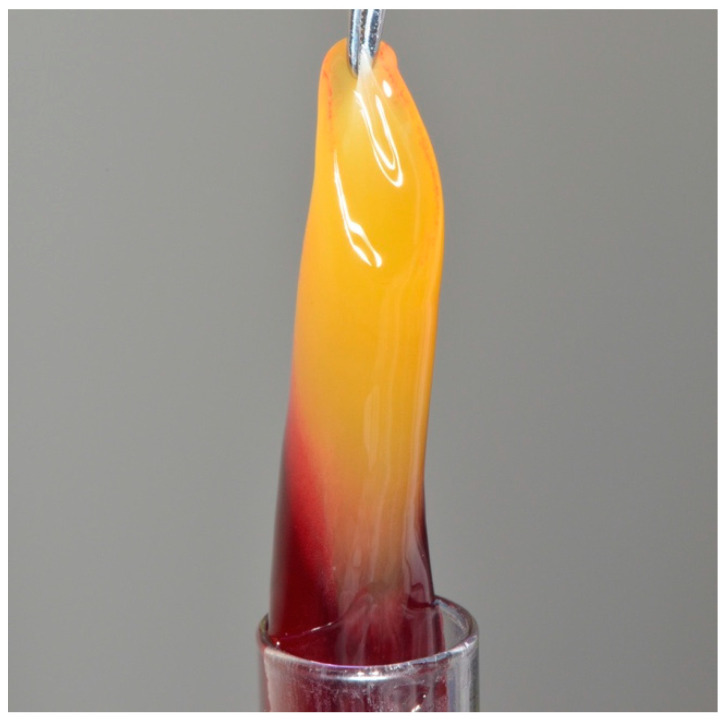
Autogenous-oriented PRF membrane.

Autogenous-originated membranes have also been reported in the literature for ARP procedures. L-PRF membranes have been utilized with promising results on bone dimension retention and the healing of the soft tissue [35,36]. An image of a PRF membrane is represented in Figure 5. The wide variety of materials used in ARP and the heterogeneity of studies does not currently allow for a conclusion regarding which barrier membrane, material or technique is superior as the gold standard for this procedure [37,38]. 

### 3.2. Horizontal Ridge Augmentation

Since the development of guided bone regeneration (GBR), the use of endosseous implants for jaw rehabilitation has also been extended to atrophic edentulous ridges [39]. The GBR concept was introduced after the GTR principle described previously in Section 3.1 of this manuscript. In GBR, a critical step is the mechanical exclusion of undesirable cells by means of a barrier membrane that allows the ingrowth of only osteogenic cells, in other words, bone regeneration [6,40]. The GBR technique has been validated by human studies where edentulous atrophic ridges were augmented before the insertion of dental implants [41,42,43].

**Figure 6:**
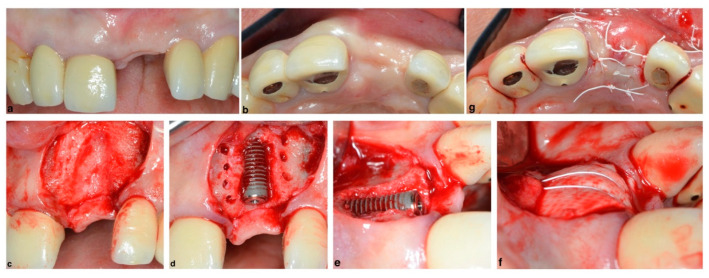
Classic horizontal ridge augmentation with a collagen resorbable membrane and xenogeneic grafting material mixed with autologous bone chips simultaneous to implant placement in the esthetic zone: (**a**) edentulous ridge, facial view; (**b**) edentulous ridge, incisal view; (**c**) soft tissue flap reflection with papilla sparing incisions where a horizontal defect was encountered; (**d**) implant placement and cortical perforation to allow for better blood supply to the graft, (**e**) horizontal ridge deficiency visible after implant placement; (**f**) bone xenograft + autologous bone chips (harvested form the nasal spine) and a resorbable collagen membrane placed and secured with strapping periosteal sutures; and (**g**) flap repositioned and secured with non-resorbable sutures.

A therapeutically oriented classification of ridge deficiency has been proposed that considers the extent and type of both bone and soft tissue resorption and consists of three main categories: (1) horizontal (H), (2) vertical (V) or (3) combination (C) defects [44]. GBR has been demonstrated to be a predictable technique regardless of the type of the defect (H,V,C), and horizontal ridge augmentations have the advantage of a high implant survival rate and a low complication rate when using resorbable membranes [45,46]. Therefore, according to the literature, the most appropriate treatment choice for horizontal ridge augmentation is a resorbable membrane in conjunction with xenogeneic particulated grafting materials, which may be mixed with autologous bone chips at a surgeon’s discretion (Figure 6) [47].

On the other hand, although they have a higher complication rate, non-resorbable ePTFE/dPTFE membranes have also been reported to be successful for horizontal GBR with a complete fill of the defect obtained more frequently compared with the sites augmented with a resorbable membrane [48]. However, the limitations of non-resorbable membranes are the increased risk of exposure and subsequent infection, the necessity for second surgery to remove them and difficult handling resulting in a technique-sensitive approach [49]. A Ti-Mesh membrane is another non-resorbable barrier membrane option. A recent systematic review showed a high success rate of implants placed either simultaneously (97%) or delayed (95.1%) [50]. However, the exposure of the membrane was present in 28% of the cases. It is important to mention that this study examined both horizontal and vertical ridge augmentation, which will be further analyzed in the next section of this report. Another option for horizontal GBR with non-resorbable materials may be the use of customized CAD/CAM Ti-Mesh with or without an additional resorbable membrane [51]. The results of this technique are promising with a clear simplification of the surgical steps compared with the use of the ePTFE/dPTFE membranes; this may be the future direction for GBR [52,53]. A comparison of the properties of the available biologic membranes and adjunctive materials/agents is shown in Table 1.

### 3.3. Vertical Ridge Augmentation

Vertical bone augmentation procedures are the most challenging, and according to the literature, the appropriate membranes for GBR in these clinical cases are non-resorbable ones such as ePTFE/dPTFE or Ti-Mesh [49]. In spite of the several disadvantages reported above, the non-resorbable membranes have the advantages of high mechanical stability of the graft, optimal space maintenance and excellent biocompatibility. Autogenous bone is highly osteogenic and is considered to be the gold standard for this vertical augmentation procedure; many clinical investigators mix the autogenous graft with xenogeneic grafting material and others mix it with allografts [54].

**Figure 7:**
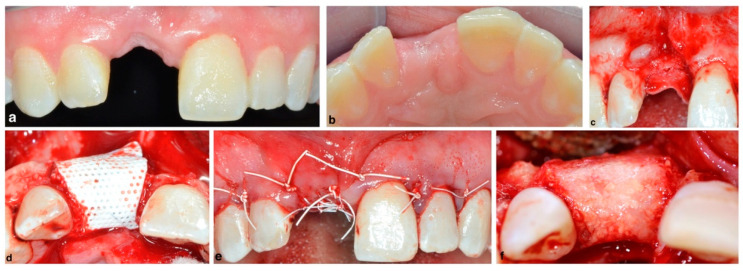
Vertical ridge augmentation with a dPTFE membrane and xenogeneic grafting material mixed with autologous bone chips simultaneous to implant placement: (**a**) pre-operative facial view of ridge deficiency in edentulous central incisor, (**b**) pre-operative incisal view of the ridge deficiency in edentulous central incisor, (**c**) soft tissue flap elevation revealing the vertical and horizontal ridge deficiency, (**d**) dPTFE non-resorbable membrane covering a mix of bone xenograft and autograft, (**e**) soft tissue flap covering the membrane and securing the flap approximation, and (**f**) 6-month post-operative ridge regeneration upon re-entry for implant placement.

Success and survival rates of implants placed in vertically augmented sites with the use of ePTFE membranes and particulated autografts yielded similar results to implants placed in native bone under loading conditions [55]. Although both ePTFE and dPTFE membranes showed identical clinical results in the treatment of vertical defects, the removal of the dPTFE membrane has been demonstrated to be easier than the ePTFE membrane [56]. A vertical GBR procedure using a dPTFE membrane is depicted in Figure 7.

Recent randomized controlled clinical trials evaluated the complication and success rates of vertical GBR utilizing non-resorbable dPTFE membranes versus Ti-Mesh with resorbable membranes [57,58]. Both approaches achieved similar results in complication rate, vertical bone gain and implant stability after rehabilitation. Customized CAD/CAM Ti-Meshes for vertical GBR resulted in a mean vertical gain of 4.78 ± 1.88 mm [53]. Although the dimensional accuracy of customized Ti-meshes needs further improvement and more comparison with the other existing approaches, it seems that the research is now focusing on this ultimate technology for vertical GBR [59].

### 3.4. Sinus Floor Augmentation

The maxillary sinus floor augmentation (SFA) procedure, commonly referred to as “sinus lift” is performed to elevate the sinus membrane, or Schneiderian membrane position. The SFA procedure is currently a widespread technique for ensuring adequate vertical height for implant placement in cases where there is a vertical ridge deficiency due to sinus pneumatization and/or ridge resorption after an extraction [60]. This SFA procedure has its origin in the late 1800s, and was established as a technique in dentistry to accommodate implant placement in the 1970s and 1980s [61,62,63,64].

**Figure 8:**
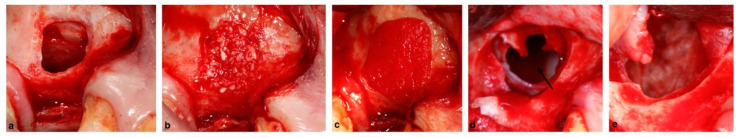
Use of resorbable membranes in SFA procedures: (**a**) SFA procedure lateral window access for membrane elevation, (**b**) freeze dried bone allograft in place after the membrane elevation, (**c**) SFA window covered with resorbable collagen membrane after bone graft placement, (**d**) Schneiderian membrane perforation during an SFA procedure (indicated by arrow), and (**e**) Schneiderian membrane perforation repaired with resorbable collagen membrane.

The use of membranes in an SFA procedure lies with the coverage of the lateral window in the direct or lateral approach and also, if they occur, in the repair of Schneiderian membrane perforations. The use of a resorbable collagen membrane in an SFA procedure is shown in Figure 8. Histologic results suggest that the presence of a membrane to cover a window leads to more vital bone formation [65]. Results from the same study show a similar percentage of vital bone formation using a resorbable or a non-resorbable membrane. Contradicting the previous study, a more recent meta-analysis that evaluated studies which investigated the presence of a membrane to cover the lateral window found no difference in vital bone formation when a membrane was absent [66]. Recently, several animal studies investigated the omission of a membrane in favor of cortical plate repositioning resulting in mixed outcomes [67,68].

Schneiderian membrane perforation is listed as one of the most commonly occurring complications in sinus floor elevation procedures. It may be dependent on the anatomy and morphology of the sinus, potential previous pathology, surgical technique and instrument or devices used [69,70,71]. Depending on the location of the perforation different treatments may be required, but in most cases, the membrane perforation requires repair via a collagen-based material, commonly a resorbable collagen membrane [72]. An alternative material is once again PRF fibrin membranes, which may be used to cover the sinus membrane perforation or the lateral window itself [73].

## 4. Discussion

Regeneration procedures are one of the greatest challenges from a wide spectrum of procedures a clinician may take on, and there are many commercially available membranes in the market, resorbable and non-resorbable. 

The current literature does not necessarily recommend a protocol or membrane type for each procedure. Regeneration procedures of osseous defects and available membrane types with supporting references are shown in Table 2. GTR is a procedure that most commonly includes a biologic membrane and bone augmentation material, with or without biologic materials. In our clinical experience, when a biologic membrane is included, the use of a resorbable membrane is most appropriate as the periodontal defects structure rarely requires the structural properties of a non-resorbable membrane; moreover, avoidance of a second surgery is beneficial in most cases.

Achieving a favorable result using the ARP procedure is less challenging than other procedures described in this manuscript. It is the clinician’s decision to select the materials for the procedure, and the authors recommend considering the defect regeneration difficulty level, as well as the cost of the materials. With SFA procedures, collagen membranes are almost exclusively used to avoid complications and the need for a second surgery.

Ridge augmentation is a procedure that always requires a biologic membrane. In a horizontal ridge augmentation, when a resorbable membrane may be selected (if the defect is simple enough to regenerate) it should be preferred to avoid the unnecessary complications and the need for removal. In vertical ridge augmentation, a non-resorbable membrane is most preferred due to the difficulty in regenerating a ridge’s height. The importance of and procedures where biocompatible membranes are used in periodontal/oral surgery regenerative treatment is shown in Table 3.

Future directions could lie in two areas: improvement of the membrane itself or investigations with specific study designs to identify the gold standard for these procedures utilizing membranes. In general, the membrane should ideally be resorbable; however, for some osseous defects, resorbable membranes may lack the necessary structural properties. On the other hand, non-resorbable membranes are more prone to infections. Treatment of the membrane with agents that improve resistance to infections would be beneficial, as well as treatment with growth factors that are known to improve soft tissue healing. Polymeric membrane treatments with antibiotics are primarily featured in non-clinical studies, making the clinical relevance of results controversial [74]. Finally, the technology of CAD/CAM printed Ti-mesh membranes is currently rapidly improving and has the potential to simplify all ridge augmentation procedures [59]. Currently, guidelines for which membrane is the ideal selection for each clinical case do not exist, therefore the clinician must make the decision based on the difficulty level of the case and their clinical experience [75,76].

According to a recent review, a more ideal membrane based on improved technology is close to becoming available [77,78]. Firstly, it is essential for the membrane to be resorbable in order to avoid a second surgical removal procedure. This membrane should be active and evaluated in its nano-structure, physical, chemical and nano-mechanical properties. Additional important attributes of an improved membrane would be its bioactivity, enhancement of cell adhesion, proliferation, differentiation by osteoblasts, as well as mineralization. Immunomodulation testing has been reported to promote macrophage recruitment, as well as the M2 osteoblast phenotype.

## 5. Conclusions

In conclusion, the practice of regeneration is challenging, and the most applicable materials are essential for successful results. Membranes may be utilized in a series of procedures based on the principles of regeneration: from small procedures such as GTR and ARP to more extensive ones such as GBR and SFA. A wide variety of membranes exist, from well-studied membranes such as PTFE and collagen membranes, to very promising ones for the future, such as Ti-Mesh membranes and PRF membranes. 

Case selection is critical, as well as knowledge of the properties of each membrane and the difficulty level of the case. It is the opinion of the authors that for cases where there is no evident benefit of using a non-resorbable membrane, a resorbable membrane should be used to eliminate the need of a second surgical procedure for removal of the membrane, which would decrease morbidity and increase patient satisfaction. However, challenging cases may benefit from the use of non-resorbable membranes with more stable physical properties, leading to a more successful result.

## Figures and Tables

**Table 1:** Properties of biologic membranes and adjunctive materials/agents.

Membranes Type	Advantages	Disadvantages
Non-resorbable membrane	Structural properties Biocompatible	More sensitive to infection Requires additional surgical procedure
Resorbable membrane	Biocompatible & degradable Single procedure/patient comfort Cost reduction	Weaker structure
Titanium mesh	Excellent structural integrity Digital printing option for custom-fitted membrane	More sensitive to infection Requires additional procedure Technique sensitive

**Table 2:** Regeneration procedures and available membrane types and materials.

Treatment	Membrane Type	Reference
Guided Tissue Regeneration	ePTFE/dPTFE Collagen Amniotic Calcium sulfate PRF EMD	[9,11,12,18,19] [10,11,13,18,19] [14,15] [12] [13,14] [13,14,16,17,18,19]
Alveolar Ridge Preservation	Collagen dPTFE PRF	[23,24,27,28,29,34,37,38] [25,27,28,30,31,32,33,34,38] [24,27,35,36]
Horizontal Ridge Augmentation	Collagen dPTFE Ti-mesh	[45,46,47,48,51] [41,42,43,46,47,48] [51,52,53]
Vertical Ridge Augmentation	ePTFE/dPTFE/Ti- mesh (with/without collagen membrane)	[55,56,57,58,59]
Sinus Floor Augmentation Window coverage Schneiderian membrane perforation repair	Collagen/ePTFE PRF Collagen PRF	[65,66,67,68] [73] [72] [73]

Abbreviations: ePTFE—expanded polytetrafluoroethylene, dPTFE—dense polytetrafluoroethylene, PRF—platelet-rich fibrin, EMD—enamel matrix derivative.

**Table 3:** The Importance of and procedures where biocompatible membranes are used in periodon-tal/oral surgery regenerative treatment.

Procedure	Desired Clinical Result	Reference
Regeneration of Intra-bony Defects	Bone augmentation and clinical attachment level gain in sites with intra-bony defects, to improve prognosis of a tooth or implant	[4,6]
Regeneration of Furcation Defects	Bone augmentation and clinical attachment level gain, in order to improve or eliminate the horizontal and vertical component of a furcation defect	[8]
Alveolar Ridge Preservation	Placement of bone graft in socket after extraction to preserve and augment existing bone for placement of future implant or preserve the alveolar ridge for a fixed bridge	[23,24]
Horizontal Ridge Augmentation	Augment horizontal width of a deficient alveolar ridge to allow implant placement	[42,47]
Vertical Ridge Augmentation	Augment vertical height of atrophic alveolar ridge to allow implant placement	[55,56]
Sinus Floor Augmentation	Augmentation of the floor of the maxillary sinus to obtain adequate vertical height for implant placement	[63,64,65,72]
